# Acquired Methemoglobinemia Leading to Death After Fungicide Ingestion: A Case Report

**DOI:** 10.7759/cureus.84086

**Published:** 2025-05-14

**Authors:** Namrata Mathur, Rashmi Ranjan Guru, Soujanya Kumar, Peter Jiss V, Sumithran N, Amit Rohila, Meenakshi Sharma, Subhodip Mitra

**Affiliations:** 1 Emergency Medicine, All India Institute of Medical Sciences, Jodhpur, Jodhpur, IND; 2 Hospital Administration, All India Institute of Medical Sciences, Jodhpur, Jodhpur, IND; 3 Hospital Administration, Symbiosis International University, Pune, IND; 4 Hospital Administration, Post Graduate Institute of Medical Education and Research, Chandigarh, IND; 5 Hospital Administration, Government Medical College Anantapur, Anantapur, IND; 6 Nursing, Post Graduate Institute of Medical Education and Research, Chandigarh, IND; 7 Hospital Administration, All India Institute of Medical Sciences, Kalyani, Kalyani, IND

**Keywords:** fungicide poisoning, methemoglobinemia, methylene blue, plasma exchange therapy, saturation gap

## Abstract

We described a patient who presented with ingestion of fungicide "Perfect Azole," which contains 7.1% azoxystrobin and 11.9% propiconazole. The methemoglobin (MetHb) level was found to be 80% in the blood. Cyanosis, which was not responding to supplemental oxygen, chocolate-brown blood, and an oxygen saturation gap of >5% raised the suspicion of methemoglobinemia. Initially, the patient was treated with the injection of methylene blue (MB) and ascorbic acid. Later, due to persistent MetHb levels, plasma exchange and exchange transfusion were done.

Early suspicion of this condition based on history and non-resolving hypoxia with oxygen and classic color on blood sampling can lead to timely management, which is of paramount importance for this life-threatening condition.

## Introduction

Methemoglobinemia is a condition in which oxygen delivery to tissues decreases. In this condition, the normal ferrous form of hemoglobin gets converted to the oxidized ferric form. Normally, reduction systems in our body, mainly the cytochrome-b5-metHb reductase system and one alternative pathway, the NADPH methemoglobin reductase system, do not allow methemoglobin (MetHb) to accumulate in the system. Imbalances in these mechanisms or increased production of MetHb lead to methemoglobinemia, and based on the level of MetHb in the blood, various signs and symptoms occur. When the concentration of MetHb reaches 20%, the blood color changes to a "chocolate brown" color, which can aid in bedside diagnosis of this condition. Mild to moderate symptoms of hypoxemia develop when the level reaches 20%-50%, as the physiologic value of the methemoglobin is 0%-2%. Methemoglobinemia becomes life-threatening when the methemoglobin (MetHb) level exceeds 70%. Azole fungicides work by disrupting the fungal cell membrane by inhibiting ergosterol synthesis [1,2].

This condition can be categorized into two types: congenital and acquired. Among them, acquired type is more common and can be caused by topical anesthetic agents (e.g., benzocaine and lidocaine). Fungicides and industrial chemicals can lead to life-threatening methemoglobinemia [3-5].

With this case report of a 25-year-old female patient, we want to emphasize the possibility of methemoglobinemia in a case report presenting with a history of fungicide exposure for a favorable outcome.

## Case presentation

A 25-year-old female patient with no past comorbidities presented with a history of ingestion of unknown quantities of agrochemical fungicide "Perfect Azole," which contains azoxystrobin and propiconazole, approximately two hours before arrival at the emergency department (ED).

On day 1, she was found unresponsive on arrival, with cyanosis of both limbs and lips, tongue, and nails. Her Glasgow Coma Scale (GCS) score was 3/15, pupils were bilateral, 2 mm sluggishly reactive, heart rate was 85/minute, and blood pressure was 80/40 mmHg. Her SpO_2_ measured by pulse oximetry was 85% on 15 L of oxygen via a non-rebreather mask, and oxygen saturation (SaO_2_) was 97%. The blood sample for arterial blood gas (ABG) was chocolate brown. Cyanosis, which was not improving with high-flow oxygen, chocolate-brown-colored blood, and a saturation gap of >5% raised the possibility of methemoglobinemia secondary to ingestion of fungicide, confirmed by ABG, with MetHb levels of 80%. The patient was intubated for airway protection and mechanically ventilated in volume control mode. Gastric lavage was done. Methylene blue (MB) (50 mg) was injected intravenously at 1 mg/kg body weight and repeated after a 30-minute time interval. An injection of ascorbic acid (2 g) was given intravenously. The rest of the physical examination was regular. MetHb level assessment was done using ABG analysis, and serial estimation of MetHb and saturation gap is depicted in Table 1.

**Table 1:** Serial ABG values ABG: arterial blood gas, FiO2: fraction of inspired oxygen, pCO_2_: partial pressure of carbon dioxide, pO_2_: partial pressure of oxygen, HCO₃: bicarbonate, SaO_2_: arterial oxygen saturation, SpO_2_: peripheral oxygen saturation, Na⁺: sodium, K+: potassium, Cl-: chlorine, MetHb: methemoglobin

Parameters	Day 1, 8 am	Day 1, 3 pm	Day 1, 11 pm	Day 2, 9 am	Day 3, 8 am	Day 3, 11 am (pre-plasma exchange)	Day 3 (post-plasma exchange)	Day 4, 8 am	Day 4, 7 pm (post-exchange transfusion)	Day 5, 2 pm	Reference range
FiO2	100	100	100	100	100	100	100	100	100	100	21%-100%
pH	7.21	7.18	7.54	7.4	7.29	7.33	7.5	7.05	7.15	6.9	7.35-7.45
pCO_2_	27.8	32.5	23.7	30	32.8	36	23.7	44	39	39	35-45
pO_2_	67.6	400	384	200	493	67.6	384	352	499	407	80-100
HCO₃	11.4	12.1	20	22	15	19	24	12	13	8	22-26
SaO_2_	95	95	98	99	100	99	98.5	97	98	95	95%-100%
SpO_2_	80%	85%	88%	89%	89%	88%	90%	85%	90%	87%	95%-100%
Na⁺	136.3	143.6	132	130	140	144	132	160	150	159	135-145 mEq/L
K+	3.74	3.95	3	3.5	3	3.71	3	2.03	3.4	3.8	3.5-5.5 mEq/L
Cl-	103.6	105	104	115	117	117	104	115	117	138	96-106 mEq/L
MetHb	80.70%	33%	14%	16%	73%	76%	17%	73%	14%	33%	<1.5%
Saturation gap	15	10	10	10	11	11	8.5	12	8	8	<5%

All laboratory parameters were within normal limits, including renal function, liver function, and hematological panel (with attention to hemolysis). The urine strip test for pregnancy was needed to rule out pregnancy, as the patient was of reproductive age, and the report was negative. A non-contrast CT scan of the head was found to be normal.

On day 2, the patient's GCS score improved to E_2_V_1_M_5_, but the patient was on mechanical ventilator support, and the methemoglobin level decreased to 17%. On day 3, the patient's GCS score dropped to E_1_V_1_M_3_, and ABG suggested a MetHb level of 73%. One session of plasma exchange was done with an exchange volume of 3 L in view of refractory methemoglobinemia, but this is not recommended as first-line therapy in such cases. The repeat MetHb level was 4.4%, but the patient's sensorium did not improve. On day 4, again, the MetHb concentration increased to 76%. In view of consistent methemoglobinemia, exchange transfusion was initiated with 4 L of volume, and it successfully reduced the MetHb level to 14%. Her GCS score did not improve, and shock worsened. The patient was on maximum vasopressor (infusion of noradrenaline, vasopressin, and adrenaline) support, but the shock did not improve, and the patient suddenly went into cardiac arrest and could not be revived despite prolonged resuscitatory efforts.

## Discussion

Based on the history of exposure to fungicide and cyanosis, which did not resolve even after high-flow oxygen with a non-rebreather mask, methemoglobinemia was suspected.

When the oxygen saturation in arterial blood gas analysis (SaO_2_) is 5% greater than oxygen saturation measured through pulse oximetry (SpO_2_), it is called an oxygen saturation gap. This indicates that an abnormal form of hemoglobin is present, like methemoglobin, sulfhemoglobin, and carboxyhemoglobin. Oxygenated and deoxygenated hemoglobin absorb different wavelengths of light. A pulse oximeter works by measuring the difference between the wavelengths of light absorbed by oxygenated and deoxygenated hemoglobin. Methemoglobin has a similar absorption spectrum; that is why pulse oximeters cannot distinguish methemoglobin from oxygenated hemoglobin, resulting in falsely high SpO_2_ values [2,3].

Initial therapy for MetHb is injection of methylene blue (MB), which is given at a dose of 1-2 mg/kg body weight intravenously over 5-10 minutes. It is given at MetHb levels above 20% in arterial blood. The MetHb level should be rechecked, and MB can be repeated at the same dose. Total intravenous dosage should be below 7 mg/kg [6].

When MB is unavailable or in conditions like G-6-PD deficiency, MB is not recommended; intravenous ascorbic acid can be used in that scenario. Ascorbic acid is a water-soluble reducing agent and antioxidant. It causes direct reduction of MetHb. Ascorbic acid dosing regimen varies, ranging from 1.0 g ascorbic acid as a one-time dose to a maximum dose of 10 g ascorbic acid every six hours [7,8].

The process of plasma exchange, called plasmapheresis, has been described in some case reports as a treatment option where intravenous methylene blue is ineffective [9].

According to a systematic review, whole blood exchange therapy led to a higher survival rate of 81.6% in patients who were refractory to intravenous methylene blue. However, this therapy has its limitations, as it requires blood bank facilities, proper technique, and equipment-related challenges. Moreover, the number of sessions and the precise volume to be exchanged are not confirmed [10,11]. In this case report, although plasmapheresis and exchange transfusion therapies were given, the patient's condition did not improve.

Limitations

As the patient came with an unconscious state of mind, limited data related to poison (amount and time of poison ingestion) were obtained from the patient's family members. Diagnosis was made based on clinical suspicion and MetHb level in arterial blood gas analysis. Limited literature is available regarding this specific fungicide poisoning all over the world.

Recommendations

When a patient presents with cyanosis that does not improve with supplemental oxygen and the presence of a saturation gap, we can suspect methemoglobinemia instead of an abnormal form of hemoglobin, like methemoglobin, sulfhemoglobin, and carboxyhemoglobin, and treatment should be started immediately. Blood gas analysis with serial methemoglobin measurements is crucial for diagnosis and early treatment.

## Conclusions

Methemoglobinemia is a serious condition that needs early diagnosis and timely intervention. This case report highlighted the importance of suspecting methemoglobinemia in conditions with an oxygen saturation gap. Methylene blue remains the first-line therapy, and in refractory cases, plasmapheresis and exchange transfusion should be considered early. Further studies are required for other potential therapies. Continued education and awareness among healthcare providers are essential for early diagnosis, timely treatment, and rehabilitation.
